# Impact of smoking on choroidal microvasculature dropout in glaucoma: a cross-sectional study

**DOI:** 10.1136/bmjophth-2023-001421

**Published:** 2023-10-29

**Authors:** Takashi Nishida, Eleonora Micheletti, Kareem Latif, Kelvin H Du, Robert N Weinreb, Sasan Moghimi

**Affiliations:** Department of Ophthalmology at the Shiley Eye Institute, University of California at San Diego, La Jolla, California, USA

**Keywords:** Glaucoma, Imaging

## Abstract

**Objective:**

To investigate the effect of smoking on choroidal microvasculature dropout (MvD) in glaucoma.

**Design:**

Cross-sectional study.

**Setting:**

Tertiary glaucoma centre.

**Participants:**

223 eyes of 163 patients with primary open-angle glaucoma who had undergone imaging with optical coherence tomography angiography and completed a questionnaire on smoking from the Diagnostic Innovations in Glaucoma Study.

**Primary outcome measures:**

Linear mixed-effects models were used to determine the effect of each parameter on MvD area and angular circumference. The sensitivity analysis was performed by categorising the glaucoma severity determined by visual field mean deviation (MD).

**Results:**

MvD was found in 37 (51.4%) eyes with smoking history and in 67 (44.4%) eyes with non-smokers (p=0.389). Larger MvD area and wider angular circumference were found in smokers compared with non-smokers (p=0.068 and p=0.046, respectively). In a multivariable model, smoking intensity was significantly associated with MvD area (0.30(95% CI 0.01 to 0.60) each 0.01 mm^2^ per 10 pack-years; p=0.044). In eyes with moderate-severe glaucoma (MD <−6), smoking intensity was associated with larger MvD area (0.47 (95% CI 0.11 to 0.83) each 0.01 mm^2^ per 10 pack-years; p=0.011), whereas no significant association was found in early glaucoma (MD ≥−6) (−0.08 (95% CI −0.26 to 0.11), p=0.401).

**Conclusions:**

Smoking intensity was associated with larger choroidal MvD area in eyes with glaucoma, especially in patients with more severe disease.

**Trial registration number:**

NCT00221897.

**WHAT IS ALREADY KNOWN ON THIS TOPIC**

- Previous studies have suggested that smoking may be a risk factor for the development and progression of glaucoma. However, the association between smoking and choroidal microvasculature dropout (MvD) in patients with glaucoma, as measured by optical coherence tomography angiography, is not well established.

**WHAT THIS STUDY ADDS**

- This retrospective cross-sectional study shows that greater pack-years of smoking are associated with larger MvD area in patients with glaucoma, particularly those with moderate-severe disease. These findings suggest that smoking may contribute to the progression of glaucoma through its effect on choroidal microvasculature.

**HOW THIS STUDY MIGHT AFFECT RESEARCH, PRACTICE OR POLICY**

- These findings highlight the potential role of smoking in the pathogenesis of glaucoma. Future research could explore the mechanisms underlying the association between smoking and MvD in patients with glaucoma and investigate the efficacy of smoking cessation interventions in patients with glaucoma.

## Introduction

Cigarette smoking has been regarded as a risk factor for the incidence and progression of several ocular disorders, such as age-related macular degeneration, cataract and retinal vein occlusion.^1–4^ However, the role of cigarette smoking in glaucoma remains uncertain. Several cohort studies found no association or a protective effect of smoking on open-angle glaucoma (OAG),^5–7^ whereas others have reported an increased risk of OAG development and progression.^8–11^

Glaucoma is characterised by loss of retinal ganglion cells which results in progressive functional loss with visual ﬁeld (VF) deterioration. Both mechanical and vascular factors contribute to the development and worsening of this condition.^12–14^ Specifically, low ocular perfusion pressure has been linked to heightened risk of glaucoma development and progression,^15^ suggesting that irregularities in microvessels may play a role in the pathophysiology of glaucoma. Choroidal microvasculature dropout (MvD), a localised parapapillary perfusion defect, has been recognised in patients with glaucoma.^16–18^ MvD, identified using optical coherence tomography angiography (OCTA), corresponds to perfusion defects on indocyanine green angiography,^17^ indicating that MvD is a valid marker for deep tissues of the optic nerve head (ONH).

MvD has been reported to be associated with glaucoma severity.^18 19^ Moreover, several studies found an association between MvD area and glaucoma progression as indicated by faster VF loss, retinal nerve fibre layer (RNFL) thinning and ganglion cell complex thinning.^18 20 21^ While a recent study found that pack-years of smoking was associated with reduced ONH vessel density, the literature about the adverse effects of the smoking on deeper ONH microvasculature is sparse. In this study, we examined the effect of smoking on choroidal MvD in patients with glaucoma.

## Methods

### Study design and participants

This was a cross-sectional study of patients with primary OAG (POAG) selected from Diagnostic Innovations in Glaucoma Study (DIGS)^22 23^ who underwent OCTA (AngioVue; Optovue, Fremont, California, USA) imaging. Longitudinal assessments were conducted based on a standardised protocol, which included periodical clinical evaluations, imaging and functional tests. Inclusion and exclusion criteria, detailed subsequently, were applied to select participants from the DIGS cohort.

Self-history of tobacco consumption was recorded. When applicable, the pack-year index was used to quantify smoking intensity, defined as the consumption of 20 cigarettes daily for 1 year. Due to limited sample size (n=2), current smokers were excluded. The latest good-quality OCTA scan was chosen for analysis for each participant. Eyes were categorised as glaucomatous based on repeatable abnormal VF test results (at least two consecutive) with evidence of glaucomatous optic neuropathy—defined as excavation, the presence of focal thinning, notching of neuroretinal rim, or localised or diffuse atrophy of the circumpapillary RNFL. An abnormal VF test was defined as a pattern SD outside of the 95% normal confidence limits or a Glaucoma Hemifield Test result outside normal limits.

Inclusion criteria also included (1) older than 18 years of age, (2) open angles on gonioscopy, and (3) best-corrected visual acuity of 20/40 or better at study entry. Exclusion criteria were (1) history of trauma or intraocular surgery (except for uncomplicated cataract surgery or glaucoma surgery), (2) coexisting retinal disease, (3) uveitis, (4) non-glaucomatous optic neuropathy, (5) axial length of 27 mm or more. Participants with the diagnosis of systemic diseases such as Parkinson’s disease, Alzheimer’s disease, dementia or a history of stroke were also excluded.

### Choroidal MvD detection

OCTA scans (4.5×4.5 mm^2^, 304×304 pixels) centred on ONH were acquired using the AngioVue OCTA system (software V.2018.1.1.63). Automated segmentation was performed by the AngioVue software, and en-face choroidal vessel density maps were generated. Quality control followed the University of California, San Diego, Imaging Data Evaluation and Analysis Reading Center guidelines. Scans of subpar quality were re-evaluated, and exclusion criteria were applied as detailed. Trained observers reviewed scans and those with poor quality were reviewed for quality. Exclusion criteria were as follows: (1) scan quality <4, (2) poor clarity, (3) residual motion artefacts visible as irregular vessel pattern or disc boundary on the en-face angiogram, (4) image cropping or local weak signal resulting from vitreous opacity, or (5) uncorrected segmentation errors. As a result, only clear images were analysed that had scan quality greater than four and also did not exhibit blurring attributable to motion or other artefacts. To meet the criteria for dropout, the following conditions had to be met: the dropout had to span at least four consecutive horizontal B-scans, have a diameter greater than 200 µm in at least one scan and make contact with the boundary of the optic disc as delineated by OCT. The software automatically identified the optic disc boundary. If inaccuracies in the boundary identification occurred, a trained observer blinded to the participants’ clinical details manually corrected the boundary by locating the position of Bruch’s membrane opening.^24^

### Assessment of choroidal MvD area, circumferential angle and location

The boundaries of the optic disc and peripapillary atrophy were identified by concurrently examining stereoscopic optic disc photographs and scanning laser ophthalmoscopic-like images acquired alongside the OCTA images. MvD was characterised as a total absence of choroidal microvasculature adjacent to the optic disc margins, with a diameter of at least 200 µ (online supplemental figure 1), a method that was previously validated.^16^ The area of MvD was manually outlined on en-face choroidal vessel density maps using ImageJ software (V.1.53). Trained graders (EM, KL), blinded to the patients’ clinical information, evaluated the presence, area and angular circumference of the MvD. Any uncertainty was resolved through adjudication with a glaucoma specialist (TN). In the current study, when assessing the MvD area on en-face images of the choroidal layer, we incorporated the region occupied by large retinal vessels as part of the MvD area, provided that the MvD extended beyond the vessels. Conversely, if the MvD did extend beyond the vessels, we excluded the portion covered by retinal vessels by quantifying the shadow area and deducting it from the MvD area measurement. Where retinal vessels were positioned at the MvD boundary, we omitted the area covered by these vessels from the MvD measurement. For MvD angular circumference, the two points at which the extreme borders of MvD area met the ONH border were identified and defined as angular circumferential margins, then determined by drawing two lines connecting the ONH centre to the angular circumference margins of the MvD.^25^ For eyes displaying multiple MvD regions, each region’s area and angular extent were calculated individually and subsequently summed to obtain the total MvD area and angular range of the eye. Moreover, the presence of large vessels or disc haemorrhages could make projection artefact on en-face choroidal vessel density images, complicating the delineation of MvD boundaries. To mitigate this issue, MvD was characterised using a standardised approach that incorporated both en-face and horizontal B-scan images. Ocular magnification was adjusted using Littmann’s formula.^26 27^ The Avanti OCT system comes with a preset axial length of 23.95 mm and an anterior corneal curvature radius of 7.77 mm.

10.1136/bmjophth-2023-001421.supp1 Supplementary data

### Statistical analysis

Patient and eye characteristics data were presented as mean (95% CI) for continuous variables and count (%) for categorical variables. Categorical variables were compared using the Χ^2^ test. Univariable and multivariable linear mixed-effects models were used to determine the effect of smoking intensity on MvD area and angular circumference. The covariate including age, sex, self-reported race, VF mean deviation (MD) and any other variable in which the p value was <0.1 in univariable analysis on the MvD area/angle was introduced in the multivariable model. The sensitivity analysis was performed by categorising the glaucoma severity determined by VF MD. Analyses of data were conducted with Stata V.16.0 (StataCorp, College Station, Texas, USA). A two-sided p<0.05 was considered statistically significant.

## Results

After exclusion of 12 eyes due to poor image quality, 223 eyes of 163 patients with POAG were included in the analysis. Demographic and baseline clinical characteristics of the participants are presented in table 1. Of them, 55 patients (33.7%) had the history of tobacco consumption (21.8% of 0–10 pack-years, 21.8% of 10–20 pack-years and 56.4% of >20 pack-years).

**Table 1:** Demographics and baseline clinical characteristics of the patients with glaucoma

Characteristic	n=223 eyes of 163 patients
Age, years	73.0 (71.4 to 74.6)
Sex, female (%)	88 (54.0)
Race (African American/non-African American)	34/129
Ever reported tobacco consumption, n (%)	55 (33.7)
>0–10 pack-years	12 (21.8)
>10–20 pack-years	12 (21.8)
20+ pack-years	31 (56.4)
Smoking intensity among ever smokers, pack-year	27.4 (21.9 to 32.9)
Hypertension, n (%)	89 (54.6)
Diabetes, n (%)	29 (17.8)
Disease severity defined by VF MD	
Early glaucoma (MD ≥−6 dB), eye no (%)	119 (53.4)
Moderate and advanced glaucoma (MD <−6 dB), eye no (%)	104 (46.6)
Presence of choroidal MvD, eye no (%)	103 (46.2)
Axial length, mm	24.4 (24.2 to 24.6)
CCT, µm	535.8 (530.1 to 541.5)
IOP, mm Hg	14.0 (13.2 to 14.7)
24-2 VF MD, dB	−7.6 (−8.4 to −6.7)
Average SSI	58.7 (57.5 to 59.8)

Values are shown in mean (95% CI), unless otherwise indicated.

CCT, central corneal thickness; IOP, intraocular pressure; MD, mean deviation; MvD, microvasculature dropout; SSI, Signal Strength Index; VF, visual field.

Table 2 shows the characteristics of eyes categorised by smoking history. Higher proportion of moderate and advanced glaucoma was found in eyes with the history of smoking compared with eyes with no history of smoking (p=0.004). Mean 24-2 VF MD was −8.9 dB in eyes with the history of smoking and −6.9 dB in eyes with no history of smoking (p=0.053). MvD was found in 37 (51.4%) eyes with the history of smoking and in 67 (44.4%) eyes with no history of smoking (p=0.389). In eyes with MvD, both the area and angular circumference of dropout were larger in patients with the history of smoking compared with those with no history of smoking (p=0.068 for MvD and p=0.046 for MvD angular circumference, respectively).

**Table 2:** Characteristics of eyes categorised by smoking history

	Smoking history (+)	Smoking history (−)	P value
Characteristic	n=72 eyes of 56 patients	n=151 eyes of 107 patients	
Age, years	73.8 (71.2 to 76.4)	72.6 (70.6 to 74.6)	0.485
Sex, female (%)	25 (44.6)	63 (58.9)	0.099
Race (African American/non-African American)	10/46	24/83	0.548
Hypertension, n (%)	37 (66.1)	52 (48.6)	**0.046**
Diabetes, n (%)	8 (14.3)	21 (19.6)	0.519
Disease severity by VF MD			**0.004**
Early glaucoma, eye no (%)	28 (38.9)	91 (60.3)	
Moderate and advanced glaucoma, eye no (%)	44 (61.1)	60 (39.7)	
Presence of choroidal MvD, eye no (%)	37 (51.4)	67 (44.4)	0.389
Choroidal MvD area among eyes with MvD, mm^2^	0.33 (0.24 to 0.43)	0.24 (0.20 to 0.28)	0.068
Choroidal MvD angle among eyes with MvD, degree	99.0 (82.5 to 115.5)	75.7 (66.2 to 85.1)	**0.046**
Axial length, mm	24.4 (24.1 to 24.7)	24.4 (24.2 to 24.6)	0.942
CCT, µm	536.2 (527.1 to 545.4)	535.6 (528.2 to 543.0)	0.927
IOP, mm Hg	14.5 (12.8 to 16.1)	13.7 (12.9 to 14.5)	0.431
24-2 VF MD, dB	−8.9 (−10.6 to −7.3)	−6.9 (−7.9 to −5.9)	0.053
Average SSI	58.4 (56.6 to 60.3)	58.8 (57.4 to 60.2)	0.779

Values are shown in mean (95% CI), unless otherwise indicated. Bold text indicates a statistically significant difference with a p value less than 0.05.

CCT, central corneal thickness; IOP, intraocular pressure; MD, mean deviation; MvD, microvasculature dropout; SSI, Signal Strength Index; VF, visual field.

**Figure 1:**
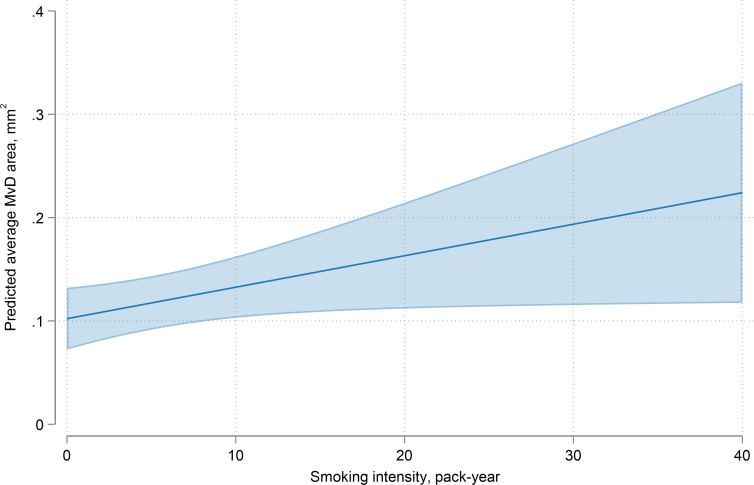
Trajectories of predicted average MvD area according to the smoking index. Other covariates were set to their mean values. The shaded area corresponds to the 95% CIs of the regression slope. MvD, microvasculature dropout.

Table 3 summarises the factors correlated with MvD area, in univariable and multivariable models. In multivariable model adjusted for age, sex, race, self-reported diabetes and VF MD, greater smoking intensity was associated with larger MvD area (0.30 (95% CI 0.01 to 0.60) each 0.01 mm^2^ per 10 pack-years; p=0.044). Figure 1 illustrates the trajectories of MvD area according to the smoking index. Online supplemental table 1 shows that smoking index was associated with MvD angular circumference in a univariable model, while not in a multivariable model (7.69 (95% CI 2.06 to 13.32) degree per 10 pack-years, p=0.008; 0.57 (95% CI −0.02 to 1.17) degree per 10 pack-years, p=0.059, respectively).

**Table 3:** Factors correlated with choroidal MvD area by univariable and multivariable linear mixed analyses

Variables	Univariable model		Multivariable model	
	Coefficient (95% CI)	P value	Coefficient (95% CI)	P value
Age (year) per 10 years	0.19 (−2.57 to 2.96)	0.890	0.13 (−2.65 to 2.91)	0.927
Sex: male	0.22 (−5.56 to 6.00)	0.940	−2.62 (−8.14 to 2.89)	0.349
Race: African American	−0.49 (−8.17 to 7.18)	0.899	2.16 (−5.81 to 10.13)	0.594
Hypertension	0.90 (−6.73 to 4.92)	0.760		
Diabetes	−7.07 (−12.63 to −1.51)	**0.013**	−6.45 (−12.87 to −0.03)	**0.049**
Axial length, per 1 mm longer	1.80 (−0.52 to 4.13)	0.127		
CCT, per 1 µm thinner	0.03 (−0.04 to 0.10)	0.414		
IOP, per 1 mm Hg higher	0.02 (−0.36 to 0.41)	0.902		
24-2 VF MD, per 1 dB worse	0.66 (0.31 to 1.01)	**<0.001**	0.54 (0.21 to 0.87)	**0.001**
Average SSI, per 1 higher	−0.01 (−0.30 to 0.27)	0.937		
Smoking intensity, per 10 pack-years	3.25 (0.47 to 6.03)	**0.022**	0.30 (0.01 to 0.60)	**0.044**

The value of MvD area was multiplied by 100 to enhance the readability of the table (unit for the coefficient is 0.01 mm^2^). Values are shown in mean (95% CI), unless otherwise indicated. Bold text indicates p value with <0.05.

CCT, central corneal thickness; IOP, intraocular pressure; MD, mean deviation; MvD, microvasculature dropout; SSI, Signal Strength Index; VF, visual field.

In eyes with moderate-severe glaucoma (VF MD <−6, n=104), smoking intensity was associated with larger MvD area (0.47 (95% CI 0.11 to 0.83) each 0.01 mm^2^ per 10 pack-years; p=0.011, table 4), whereas no significant association was found in early glaucoma (VF MD ≥−6, n=119) (−0.08 (95% CI −0.26 to 0.11), p=0.401, online supplemental table 2).

**Table 4:** Factors correlated with choroidal MvD area by univariable and multivariable linear mixed analyses in eyes with moderate to advanced glaucoma (n=104)

Variables	Univariable model		Multivariable model	
	Coefficient (95% CI)	P value	Coefficient (95% CI)	P value
Age (year) per 10 years	1.55 (−2.95 to 6.05)	0.495	0.49 (−3.89 to 4.86)	0.825
Sex: male	−4.17 (−13.82 to 5.47)	0.392	−8.96 (−17.97 to 0.05)	0.051
Race: African American	3.14 (−10.33 to 16.6)	0.644	6.32 (−7.82 to 20.46)	0.377
Hypertension	−1.60 (−11.47 to 8.27)	0.748		
Diabetes	−14.77 (−22.47 to −7.08)	**<0.001**	−4.64 (−14.31 to 5.03)	0.342
Axial length, per 1 mm longer	1.77 (−1.57 to 5.10)	0.294		
CCT, per 1 µm thinner	0.05 (−0.07 to 0.17)	0.383		
IOP, per 1 mm Hg higher	0.25 (−0.33 to 0.83)	0.386		
24-2 VF MD, per 1 dB worse	0.14 (−0.48 to 0.77)	0.655	0.17 (−0.51 to 0.84)	0.623
Average SSI, per 1 higher	0.16 (−0.41 to 0.73)	0.583		
Smoking intensity, per 10 pack-years	3.77 (0.38 to 7.17)	**0.030**	0.47 (0.11 to 0.83)	**0.011**

The value of MvD area was multiplied by 100 to enhance the readability of the table (unit for the coefficient is 0.01 mm^2^). Values are shown in mean (95% CI), unless otherwise indicated. Bold text indicates p value with <0.05.

CCT, central corneal thickness; IOP, intraocular pressure; MD, mean deviation; MvD, microvasculature dropout; SSI, Signal Strength Index; VF, visual field.

## Discussion

The present study demonstrated that greater smoking intensity was associated with larger choroidal MvD area in eyes with glaucoma. The area of dropout is larger in heavy smokers with moderate-severe glaucoma, whereas no association was found in eyes with early glaucoma.

A prior study has shown that greater pack-years of smoking were associated with faster rates of RNFL thinning in glaucoma eyes, especially when smoking intensity was higher than 8 pack-years.^10^ In another study, heavy smokers showed higher risk of sustaining VF loss over 12 years of follow-up, corroborating the idea that extent of smoking could be an important factor in the progression of glaucoma.^11^

Several lines of evidence suggest that MvD may be deleterious to glaucoma. Systemic vascular risk factors, such as diastolic blood pressure, cold extremities and migraine, which have been previously associated with pressure-independent OAG, have been found in eyes with MvD. Furthermore, MvD was more commonly found in eyes with disc haemorrhage, which have been associated with faster VF loss. In the current study, smoking intensity was associated with MvD area, suggesting that having an intense history of tobacco consumption may affect the choroidal and deep optic nerve microvasculature. Our findings are in agreement with a previous study, in which the pack-years of smoking was associated with lower ONH capillary density, as measured by OCTA.^28^ The findings indicate that reduced optic disc blood ﬂow in individuals who smoke could exacerbate damage to the retinal nerve fibres in eyes with glaucoma, thereby increasing their vulnerability to disease progression.

A possible explanation for these findings is that smoking may affect the microcirculation through various mechanisms, such as modulating endothelial-mediated vasodilation, promoting platelet clustering, impairing endothelial cell performance and stimulating the activity of circulating leucocytes. Tobacco compounds have also been reported to reduce blood supply by nicotine-induced vasospasm, atherosclerotic narrowing of capillary vessels and thrombotic occlusions. Therefore, smoking may affect the choroidal and deep optic nerve microvasculature, extending the areas of dropout.

The UK Glaucoma Treatment Study reported a 41% lower HR for VF deterioration over a 2-year follow-up period among individuals who were current or former smokers.^7^ Similarly, data from the Nurses’ Health Study and the Health Professionals Follow-up indicated a slight negative correlation between the intensity of smoking and the incidence of glaucoma.^29^ The varying results across different studies on the relationship between smoking and glaucoma could be partially explained by the intricate nature of their interplay. Findings from the National Health and Nutrition Examination Survey suggested that current smokers had reduced odds of developing glaucoma in univariable analyses, although this relationship was not statistically significant in multivariable models.^30^ In contrast, greater smoking intensity was associated with higher odds of glaucoma among smokers. It was posited that any potential protective effects of smoking could be eliminated in individuals who smoke heavily. In the present study, as shown in figure 1, after adjusting for variables such as age, sex, race, diabetes status and 24-2 VF MD, greater pack-years of smoking were associated with a larger MvD area, supporting the above hypothesis.

To our knowledge, this research represents an initial investigation into the relationship between MvD and smoking. Prior research has shown the impact of smoking, and its intensity, on optic nerve damage in patients with glaucoma. Our study reveals more profound microvascular damage in the optic nerve’s deep tissues, especially in those with a history of smoking. The identification of these perfusion defects might help clinicians to identify patients requiring more aggressive management and smoking cessation. The present study also demonstrated that the relationship between intensity of smoking and choroidal MvD depends on the severity of the disease. Specifically, in patients with moderate-advanced glaucoma, MvD area was associated with smoking intensity, whereas this relationship was not observed in patients with early glaucoma. These findings may be related to the microvascular circulation already being severely compromised in eyes with moderate-advanced glaucoma. A history of intense tobacco consumption may have led to further deterioration of microvessels, thereby contributing to more extensive glaucomatous damage. Conversely, in eyes with mild glaucoma having healthier microvessels, smoking intensity may not induce a substantial damage to the choroidal perfusion.

This study presents several limitations. First, the sample size, especially for heavy smokers, was limited in this study. Second, although we aimed to determine the smoking intensity of the participants by providing them for completion a self-reporting form, some patients were unable to complete the form and we had to rely on asking them about their smoking habits. Self-reported data are subject to recall bias as patients may have difficulty remembering the exact amount of tobacco they consumed over a certain period. This could have resulted in underestimation or overestimation of the participants’ smoking intensity. Moreover, the questionnaire was done only once as part of the study. Participants’ smoking habits may have changed during the study period. Longitudinal data pertaining to individual tobacco purchases and usage may more accurately depict the association with the disease. Therefore, longitudinal research is required to validate the findings of the current study. Third, our study did not consider the effect of secondhand smoking on glaucoma; this is typically challenging to assess as it may vary depending on factors such as the frequency and duration of exposure, the proximity to the smoker and the ventilation in the area. Therefore, while the potential impact of passive smoking on glaucoma would be an important area of research, it may require large-scale, longitudinal studies with carefully designed exposure assessments and control for confounding factors to provide more definitive answers. Fourth, we conducted some measurements manually using Image J software. While our prior work has shown strong agreement between different examiners when measuring MvD area and angles using this approach,^18^ establishing an automated method could offer advantages, particularly for cases such as myopia or tilted optic disc. Finally, measurements taken by OCTA may vary depending on the instrument used.^31^ Therefore, it is essential to verify whether results from MvD measured using different machines can be compared externally.

In conclusion, greater smoking intensity was associated with larger choroidal MvD area in glaucoma, especially in patients with more severe disease.

## Data Availability

Data are available upon reasonable request. The datasets generated and/or analysed during the current study are available from the corresponding author on reasonable request.
