# Supplementary material for: Impact of smoking on choroidal microvasculature dropout in glaucoma: a cross-sectional study
Source: BMJ Open Ophthalmol. 2023 Oct 29;8(1):e001421. doi: 10.1136/bmjophth-2023-001421 (PMC10619022; doi:10.1136/bmjophth-2023-001421)
Supplement: Supplementary data [file bmjophth-2023-001421supp001.pdf]

Supplemental Figure 1

En-face choroidal vessel density image shows choroidal microvasculature dropout (MvD) area and angular circumference. The points where the MvD's outermost edges met the optic nerve head (ONH) were marked as angular boundaries. The angular circumference was then determined by plotting lines from the ONH center to these boundaries of the MvD.

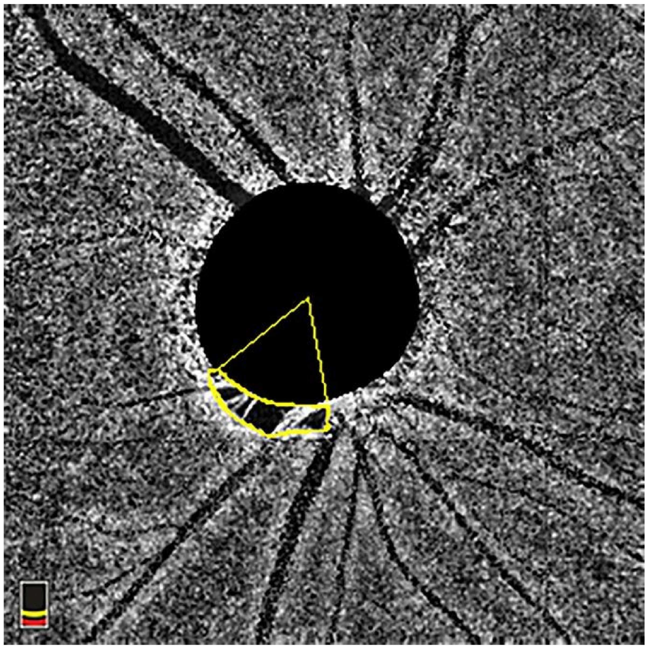

Supplemental Table 1. Factors Correlated with Choroidal MvD Angle by Univariable and Multivariable Linear Mixed Analysis (n = 223)

Variables	Univariable Model		Multivariable Model	
	coefficient, 95% CI	P value	coefficient, 95% CI	P value
Age (year) per 10 years	-1.05 (-8.53 to 6.43)	0.782	-0.86 (-7.84 to 6.12)	0.808
Sex: male	3.46 (-11.85 to 18.77)	0.656	-3.44 (-18.68 to 11.80)	0.656
Race: African American	-6.32 (-25.10 to 12.45)	0.507	-2.45 (-20.03 to 15.13)	0.784
Hypertension	0.17 (-15.11 to 15.45)	0.983		
Diabetes	-20.79 (-37.56 to -4.02)	**0.015**	-17.52 (-36.97 to 1.93)	0.077
Axial length, per 1mm longer	5.19 (-0.40 to 10.78)	0.069		
CCT, per 1 μm thinner	0.05 (-0.12 to 0.21)	0.582		
IOP, per 1 mmHg higher	0.54 (-0.68 to 1.76)	0.384		
24-2 VF MD, per 1 dB worse	3.03 (1.84 to 4.21)	**<0.001**	2.82 (1.69 to 3.95)	**<0.001**
Average SSI, per 1 higher	0.24 (-0.49 to 0.97)	0.513		
Smoking intensity, per 10 pack-years	7.69 (2.06 to 13.32)	**0.008**	0.57 (-0.02 to 1.17)	0.059

CCT = central corneal thickness; IOP = intraocular pressure; MD = mean deviation; SSI = signal strength index; VF = visual field; wiCD = whole image capillary density. Values are shown in mean (95% confidence interval), unless otherwise indicated. Bold text indicates p-value with <0.05.

Supplemental Table 2. Factors Correlated with Choroidal MvD Area by Univariable and Multivariable Linear Mixed Analysis in Eyes with Early Glaucoma (n = 119)

Variables	Univariable Model		Multivariable Model	
	coefficient, 95% CI	P value	coefficient, 95% CI	P value
Age (year) per 10 years	-0.81 (-3.99 to 2.38)	0.616	-1.29 (-4.78 to 2.20)	0.466
Sex: male	2.40 (-4.07 to 8.87)	0.464	3.38 (-3.74 to 10.51)	0.348
Race: African American	-3.63 (-10.08 to 2.82)	0.267	-3.53 (-9.67 to 2.60)	0.256
Hypertension	-1.30 (-7.59 to 5.00)	0.683		
Diabetes	-0.41 (-8.30 to 7.48)	0.918		
Axial length, per 1mm longer	1.65 (-1.28 to 4.58)	0.267		
CCT, per 1 µm thinner	0.00 (-0.07 to 0.07)	0.960		
IOP, per 1 mmHg higher	-0.18 (-0.67 to 0.30)	0.453		
24-2 VF MD, per 1 dB worse	1.72 (0.03 to3.41)	**0.046**	1.80 (-0.05 to 3.66)	0.057
Average SSI, per 1 higher	0.00 (-0.35 to 0.36)	0.987		
Smoking intensity, per 10 pack-years	-0.36 (-2.00 to 1.28)	0.665	-0.08 (-0.27 to 0.11)	0.432

The value of MvD area was multiplied by 100 to enhance the readability of the table (unit for the coefficient is 0.01 mm^2). CCT = central corneal thickness; IOP = intraocular pressure; MD = mean deviation; SSI = signal strength index; VF = visual field; wiCD = whole image capillary density. Values are shown in mean (95% confidence interval), unless otherwise indicated. Bold text indicates p-value with <0.05.
